# Influence of low-dose radiation on abscopal responses in patients receiving high-dose radiation and immunotherapy

**DOI:** 10.1186/s40425-019-0718-6

**Published:** 2019-09-04

**Authors:** Hari Menon, Dawei Chen, Rishab Ramapriyan, Vivek Verma, Hampartsoum B. Barsoumian, Taylor R. Cushman, Ahmed I. Younes, Maria A. Cortez, Jeremy J. Erasmus, Patricia de Groot, Brett W. Carter, David S. Hong, Isabella C. Glitza, Renata Ferrarotto, Mehmet Altan, Adi Diab, Stephen G. Chun, John V. Heymach, Chad Tang, Quynh N. Nguyen, James W. Welsh

**Affiliations:** 1 Department of Radiation Oncology, University of Texas M.D. Anderson Cancer Center, Unit 97, 1515 Holcombe Blvd, Houston, TX 77030 USA; 2 Department of Radiation Oncology, Shandong Cancer Hospital Affiliated to Shandong University, 440 Jiyan Road, SD, CN, Jinan, China; 3 Department of Radiation Oncology, Allegheny General Hospital, 320 East North Avenue, Pittsburgh, PA USA; 4 College of Medicine Phoenix, University of Arizona, 425 N. Fifth Street, Phoenix, AZ USA; 5 Department of Experimental Radiation Oncology, The University of Texas MD Anderson Cancer Center, 1515 Holcombe Blvd, Houston, TX USA; 6 Department of Diagnostic Radiology, The University of Texas MD Anderson Cancer Center, 1515 Holcombe Blvd, Houston, TX USA; 7 Department of Investigational Cancer Therapeutics, The University of Texas MD Anderson Cancer Center, 1515 Holcombe Blvd, Houston, TX USA; 8 Department of Melanoma Medical Oncology, The University of Texas MD Anderson Cancer Center, 1515 Holcombe Blvd, Houston, TX USA; 9 Thoracic Head and Neck Medical Oncology, The University of Texas MD Anderson Cancer Center, 1515 Holcombe Blvd, Houston, TX USA

**Keywords:** Stereotactic ablative radiation therapy, Low-dose radiotherapy, Immunotherapy, Abscopal effect, Metastatic cancer

## Abstract

**Background:**

Preclinical evidence suggests that low-dose radiation may overcome the inhibitory effects of the tumor stroma and improve a tumor’s response to immunotherapy, when combined with high-dose radiation to another tumor. The aim of this study was to evaluate tumor responses to this combination in a clinical setting.

**Methods:**

A post-hoc analysis of 3 ongoing immunoradiation trials was performed. Twenty-six (of 155) patients received low-dose radiation (1–20 Gy total), either as scatter from high-dose radiation or from intentional treatment of a second isocenter with low-dose radiation, were evaluated for response. The low-dose lesions were compared to lesions that received no radiation (< 1 Gy total). Response rates, both defined as complete and partial responses as defined by RECIST criteria were used to compare lesion types.

**Results:**

The 26 patients had a total of 83 lesions for comparison (38 receiving low-dose, 45 receiving no-dose). The average dose given to low-dose lesions was 7.3 Gy (1.1–19.4 Gy), and the average time to response was 56 days. Twenty-two out of 38 (58%) low-dose lesions met the PR/CR criteria for RECIST compared with 8 out of 45 (18%) no-dose lesions (*P* = 0.0001). The median change for longest diameter size for low-dose lesions was − 38.5% compared to 8% in no-dose lesions (*P* < 0.0001). Among the low-dose lesions that had at least one no-dose lesion within the same patient as a control (33 and 45 lesions respectively), 12 low-dose lesions (36%) responded without a corresponding response in their no-dose lesions; Conversely, two (4%) of the no-dose lesions responded without a corresponding response in their low-dose lesion (*P* = 0.0004).

**Conclusions:**

Low-dose radiation may increase systemic response rates of metastatic disease treated with high-dose radiation and immunotherapy.

**Electronic supplementary material:**

The online version of this article (10.1186/s40425-019-0718-6) contains supplementary material, which is available to authorized users.

## Introduction

Metastatic cancer has historically been considered incurable. Recent advances in immunotherapy have led to improved long-term complete responses, but only a subset of these patient see benefit. An additional proportion of patients with metastatic disease may experience systemic effects from local therapies such as stereotactic ablative radiotherapy (SABR). First described by R.H. Mole, the abscopal effect refers to an immune-mediated response of distant lesions to irradiation of other lesions; Mole considered this evidence that radiation turned lesions into “*in situ* vaccines” [1]. However, abscopal effects are quite rare in clinical practice [2], and factors that may amplify the occurrence of this phenomenon remain elusive.

Preclinical studies have suggested that low-dose radiation, although not tumoricidal on its own, may activate and stimulate immune cells and modulate the stromal microenvironment so as to facilitate the action of immunotherapy [3]. Our own post-hoc analysis of a recently completed trial of ipilimumab with high-dose radiation revealed that tumors exposed to low-dose scatter radiation (owing to their proximity to the targeted tumor) were more likely to show a response than were distant tumors exposed to no radiation [4]. From these observations, we developed a model where high-dose and low-dose radiation may work synergistically to promote systemic immunotherapy: In this model, high-dose radiation increases antigen release and presentation and primes immune cells [5], whereas low-dose radiation promotes immune-cell infiltration into the stroma and tumor bed.

Here we report on a subset of 26 patients from ongoing prospective trials of immunotherapy with radiation for metastatic cancer to further expand on our previous post-hoc analysis. These patients received low-dose radiation to metastatic lesions in combination with high-dose SABR to another lesion along with checkpoint inhibitors. We report outcomes in terms of the response of those low-dose–irradiated lesions, as well as responses of unirradiated lesions, in these patients. Our results suggest that low-dose radiation may be capable of enhancing an immune response that leads to abscopal effects.

## Methods

This post hoc analysis was reviewed and approved by the UT MDACC institutional review board. We retrospectively reviewed electronic medical records and radiation treatment plans from 155 patients enrolled and treated on our three institutional prospective clinical trials combining immunotherapy and radiation: a phase I/II “basket” trial of ipilimumab (anti-CTLA4) with SABR for patients with liver or lung metastases (NCT02239900), a phase I/II randomized trial of pembrolizumab (anti-PD1) with SABR for patients with non-small cell lung cancer (NCT02444741), and phase II “basket” trial of SABR + low-dose radiation for patients with disease progression on immunotherapy (NCT02710253); treatment took place from August 2013 through March 2019. From the datasets and radiation treatment plans of all three prospective studies, we identified 26 patients who had lesions that received low-dose radiation (“low-dose” lesions), i.e., doses of 1–20 Gy, either intentionally or unintentionally; 22 of these patients also had lesions that received < 1 Gy (“no-dose” lesions). We compared rates and extent of response of the low-dose and no-dose lesions as follows.

Lesion diameters were measured on computed tomography (CT) or positron emission tomography (PET) /CT scans of the chest, abdomen, and pelvis, and the longest diameter of each lesion were used to assess changes in lesion size. Lesion response was accessed using RECIST criteria for response, using the largest diameter of each lesion [6]. Briefly, a complete response (CR) is defined as 100% resolution of the lesion, partial response (PR) as a reduction of ≥30%, stable disease (SD) as a reduction of < 30% to an increase of < 20%, and progressive disease (PD) as an increase of > 20% in lesion size. Response was to be assessed every 3 months per specific protocol, with the same imaging modality to be used before and after treatment.

Lesions were contoured on the original treatment plan, and information on radiation doses including mean doses for each individual lesion were collected from dose-volume histograms from radiation treatment plans that had been created on a Philips Pinnacle^3^ radiation treatment planning system with the help of the study dosimetrist. All lesions and doses were approved by the treating radiation oncologist.

### Statistical analysis

The endpoint was response to low-dose radiation. The best response of each lesion was used in statistical analyses. All statistical analyses were done with SPSS v25, and graphics were produced with GraphPad Prism v8. Significance was evaluated with Fisher’s exact tests comparing no-dose lesion response groups against low-dose response groups and between specific radiation doses. Mantel-Haenzel test for independence was performed to determine whether sub-groups may be contributing to significant differences in response. Kaplan Meier survival analysis was performed to compare survival between low-dose lesion responders and non-responders.

## Results

Twenty-six patients (with 83 lesions [38 low-dose and 45 no-dose]) were evaluated in this analysis (Table 1). The most common tumor histology was adenocarcinoma (*n* = 13 [50%]), followed by squamous cell carcinoma (*n* = 3 [12%]). The most common high-dose tumor sites were lung (*n* = 17 [65%]) followed by liver (*n* = 6 [23%]). The most common sites for lesions receiving low-dose radiation were also lung (*n* = 15 [58%]) followed by liver (n = 6 [23%]) and abdomen (n = 3 [12%]).

**Table 1:** Baseline Patient and Disease Characteristics and Best Responses after Low-Dose RT

ID #	Age	Sex	Histology	IO Agent	High-Dose RT Site	High Dose RT, (Gy/fx)	Low-Dose Site	Low-Dose Type	Mean Low-Dose (Gy/fx)	Time between RT & IO	Time to Response to RT, days	Low-Dose Lesions, no.	Low-Dose Lesion Response, %*	No-Dose Lesions, no.	No-Dose Lesion Response, %*
1	91	M	Adenocarcinoma	Pembrolizumab	Lung	45/15	Lung	Scatter	18.92/15	0	42	1	−100%	2	8%
2	52	M	Adenocarcinoma	Pembrolizumab	Lung	50/4	Lung	Scatter	2.45/4	52	26	1	−19%	2	−30%
3	69	M	SCC	Ipilimumab	Lung	50/4	Lung	Scatter	6.49/4	32	54	3	−100%	1	−22%
4	21	F	HCC	Ipilimumab	Lung	50/4	Lung	Scatter	3.05/4	39	21	1	−65%	5	−38%
5	73	F	Adenocarcinoma	Ipilimumab	Lung	50/4	Lung	Scatter	2.47/4	27	42	1	−21%	1	−2%
6	67	F	Adenocarcinoma	Ipilimumab	Liver	50/4	Liver	Scatter	6.43/4	1	38	1	−59%	0	–
7	63	M	Adenocarcinoma	Ipilimumab	Liver	50/4	Liver	Scatter	9.32/4	1	35	1	−47%	1	−45%
8	60	M	Adenocarcinoma	Ipilimumab	Liver	50/4	Lung	Scatter	24.11/4	25	39	1	−63%	3	7%
9	49	F	ACC	Pembrolizumab	Lung	50/4	Lung	Scatter	12.03/4	39	38	2	−6%	1	−4%
10	53	F	Adenocarcinoma	Ipilimumab	Lung	50/4	Lung	Scatter	4.01/4	27	23	1	− 35%	2	−23%
11	44	M	ACC	Ipilimumab	Lung	50/4	Lung	Scatter	5.14/4	37	43	2	−43%	2	−33%
12	65	M	CRC	Ipilimumab	Lung	50/4	Lung	Scatter	19.43/4	29	10	2	−41%	4	44%
13	43	M	RCC	Ipilimumab	Lung	50/4	Liver	Scatter	14.4/4	1	45	1	−53%	5	−41%
14	56	M	Neuroendocrine	Ipilimumab	Liver	50/4	Liver	Scatter	15.5/4	26	153	3	−11%	3	10%
15	59	M	ACC	Ipilimumab	Liver	50/4	Liver	Scatter	21.8/4	22	12	3	−5%	3	−7%
16	74	M	Adenocarcinoma	Ipilimumab	Lung	50/4	Abdomen	Scatter	6.27/4	1	40	1	−36%	1	14%
17	62	F	Adenocarcinoma	Ipilimumab	Lung	50/4	Lung	Scatter	6.06/4	96	38	1	−42%	2	−55%
18	71	M	Adenocarcinoma	Pembrolizumab	Lung	50/4	Lung	Scatter	12.97/4	105	81	1	−100%	1	53%
19	49	M	DLBCL	Pembrolizumab	Inguinal	50/20	Inguinal	Scatter	4.74/20	31	70	1	−100%	1	−4%
20	53	M	Adenocarcinoma	Pembrolizumab	Lung	45/15	Lung	Scatter	12.20/15	74	114	1	−42%	0	–
21	53	M	SCC	Pembrolizumab	Lung	50/4	Abdomen	Intentional	8/4	39	109	1	−25%	1	−56%
22	65	F	Adenocarcinoma	Pembrolizumab	Lung	52.5/15	Lung	Intentional	7.5/5	27	119	2	−67%	1	64%
23	69	M	MCC	Atezolizumab	Adrenal	70/10	Inguinal	Intentional	6/6	27	19	2	−32%	0	–
24	80	M	SCC	Nivolizumab	Lung	52.5/15	Lung	Intentional	6/6	26	67	1	−11%	0	–
25	56	F	Melanoma	Ipilimumab	Spleen	25/5	Liver	Intentional	7.5/5	1	29	2	−7%	2	22%
26	69	F	Adenocarcinoma	Atezolizumab	Liver	60/10	Abdomen	Intentional	8/4	1	39	1	−7%	1	0%

Abbreviations: *RT* radiation, *IO* immunotherapy, *SCC* squamous cell carcinoma, *HCC* hepatocellular carcinoma, *ACC* adrenal cortical carcinoma, *RCC* renal cell carcinoma, *DLBCL* diffuse large B cell lymphoma, *MCC* Merkel Cell Carcinoma

Most patients (*n* = 20) received SABR to the high-dose targeted lesion, and the other 6 received intensity-modulated radiation (IMRT). In terms of the non-targeted lesions, 20 patients received low-dose radiation, defined as either scatter from the periphery of the high-dose field for the target lesion, and the other 6 patients received intentional low-dose radiation to 1 or more lesions in addition to lesions targeted with high-dose radiation. Ipilimumab (anti-CTLA-4) was given to 15 patients, pembrolizumab (anti-PD-1) to 8, and atezolizumab (anti-PDL1) to 2, either before or concurrent with radiation therapy. Twenty-two patients (85%) also had at least 1 lesion that did not receive any radiation (i.e., < 1 Gy), and those “no-dose” lesions were used as within-patient comparisons of response. Among those 22 patients, we compared 45 no-dose lesions against 33 low-dose lesions for this analysis.

**Fig. 1:**
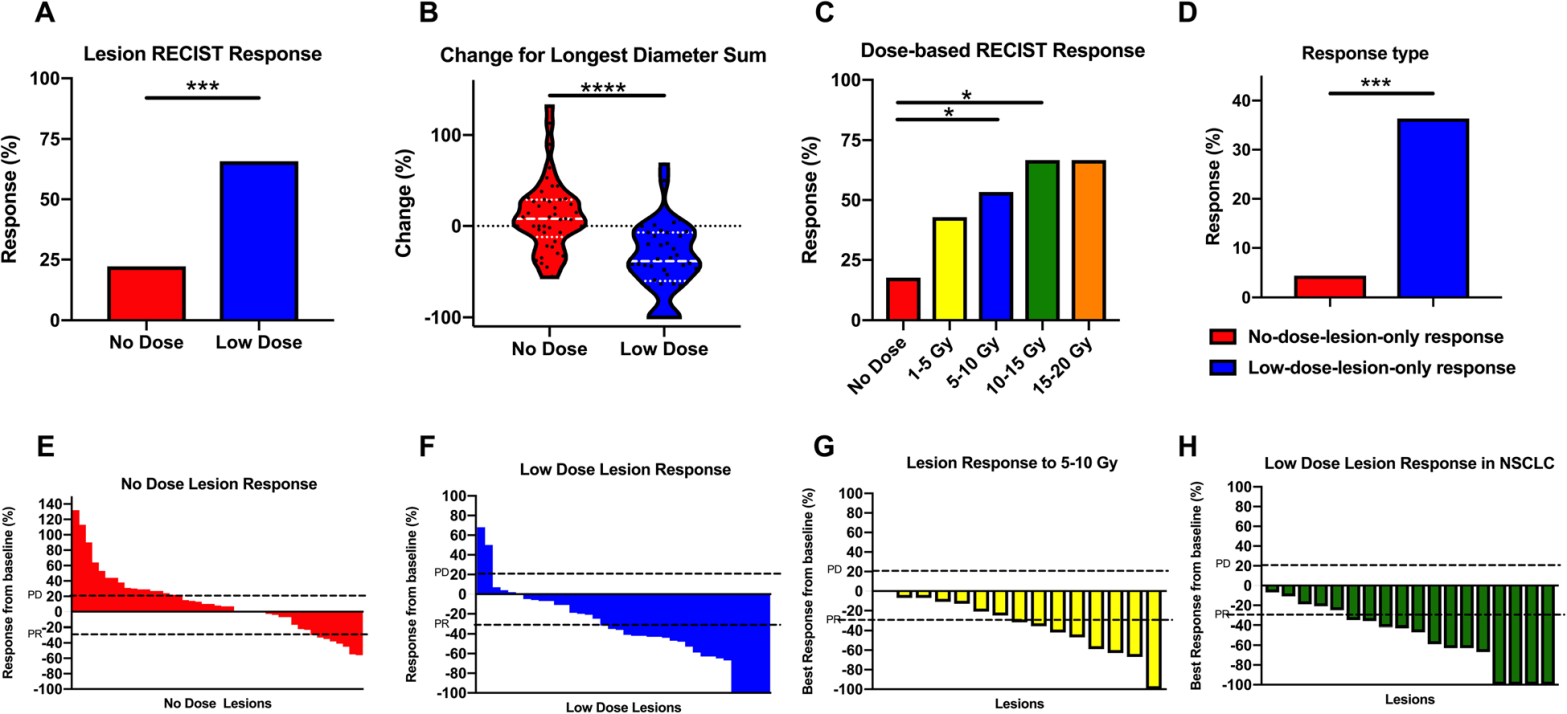
Low-dose radiation improves abscopal responses based on RECIST criteria. **a**, the percentage of lesions showing a clinical response based on RECIST criteria (CR/PR) was 53% (20 of 38) in low-dose lesions compared to 18% (8 of 45) no-dose lesions, ***P < 0.001. **b**, the median change for the sum of the longest diameter for low-dose lesions was − 38.5% (range − 100 to 68%) compared to 8% (range − 75 to 132%) in no-dose lesions, ****P < 0.0001. **c**, the percentage of lesions responding according to radiation dose. *P < 0.05. d, of the lesions from 22 patients with both no-dose (*n* = 45) and low-dose (*n* = 33) lesions, 12 lesions (36%) had low-dose-only responses at 6 months, and two (4%) had no-dose-only responses. **e**, Waterfall plot of no-dose tumor responses in patients having both lesion types. **f**, Waterfall plot of low-dose tumor responses in patients having both lesion types. **g**, Waterfall plot of low-dose tumors receiving 5–10 Gy in patients having both lesion types. **h**, Waterfall plot of low-dose tumors with NSCLC histology

In our first assessment, we asked if lesions that received low-dose radiation responded differently compared with lesions that were completely out of field. We found that 22 of 38 (58%) low-dose lesions met the PR/CR criteria for RECIST compared with 8 out of 45 (18%) no-dose lesions (*P* = 0.001) (Fig. 1a). The median change for longest diameter size for low-dose lesions were − 38.5% (range − 100 to 68%) compared to 8% (range − 75 to 132%) in no-dose lesions (*P* < 0.0001) (Fig. 1b). The mean value of the low-dose radiation (i.e., either scatter or intentional) per lesion across all 26 patients was 7.3 Gy (range 1.1–19.4 Gy). The median time between immunotherapy and radiation was 27 days (range 0-105 days), the median time between response to RT was 39.5 days (range 10–153 days) and the median time from response to immunotherapy was 58 days (range 30–218 days). All lesions that responded to low-dose radiation had maintained this response at 6 months after treatment.

We evaluated lesion response (based on RECIST criteria) in relation to radiation doses given. Significant differences in lesion responses were found for lesions treated with 5–10 Gy (*P* = 0.01), and 10–15 Gy (*P* = 0.03) (Fig. 1c). Considering 5–10 Gy dose range provided the best response, we performed a subgroup analysis to compare responses between SBRT(25Gy/5, 50Gy/4, 60Gy/10 and 70Gy/10) and traditional radiation (45Gy/15, 50Gy/20 and 52.5Gy/15). No statistical difference was found for this comparison (*P* = 0.3; Additional file 1: Figure S1A). To further explore whether sub-classifications were responsible for the enhancement of response rates observed in the 5-10Gy low-dose group, we performed a Mantel-Haenzel test for independence of the variables. No significant differences between the variables explored including age, gender, RT site, immunotherapy, and fractionation were identified (Additional file 2: Table S1). Our evaluation of the 22 patients who had both low-dose lesions (*n* = 33) and no-dose lesions (*n* = 45) showed that 12 low-dose lesions (36%) showed a low-dose-lesion-only response; by comparison, two no-dose lesions (4%) showed a no-dose-lesion-only response (*P* = 0.0004; Fig. 1d).

We also compared overall survival between those low-dose lesions which responded versus those that did not. Overall survival (OS) was found to be undefined and 53 months for responders and non-responders respectively with no statistical significance between these two groups (*P* = 0.42; HR = 0.59; 95% CI, 0.17–1.98; S Fig. 1b).

Corresponding waterfall plots demonstrate response rates were higher among low-dose lesions than among unirradiated lesions overall (Fig. 1e and f). A similar waterfall plot for lesions responding to 5–10 Gy demonstrates a RECIST criteria response rate 53% (8/15 lesions) (Fig. 1g). An additional waterfall plot was performed for just NSCLC histology which demonstrated a response rate of 72% (13/18 lesions) with 0 lesions meeting PD criteria (Fig. 1h).

### Representative cases

**Fig. 2:**
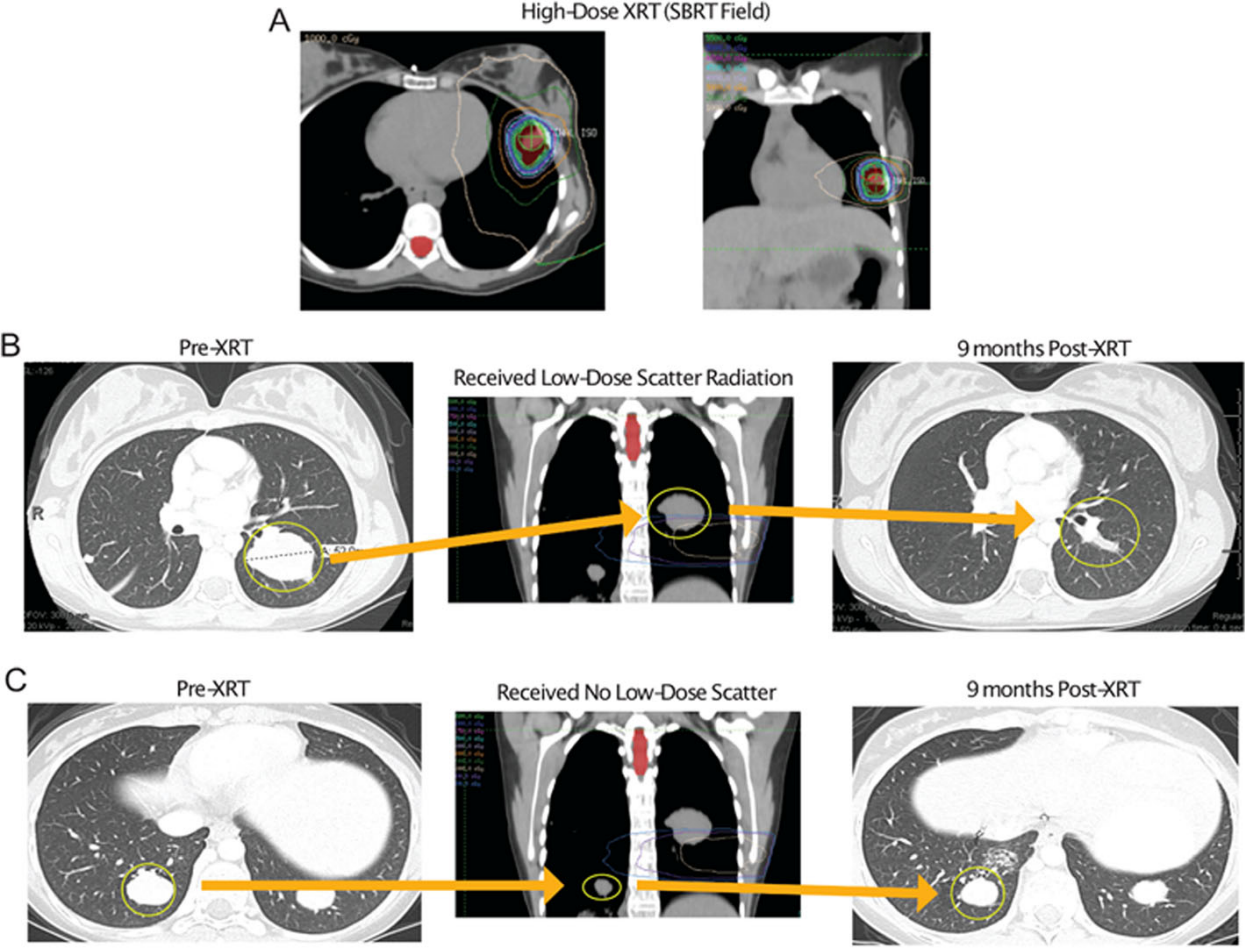
Representative scans from a patient receiving scatter radiation to a low-dose lesion. Scans from a 20-year-old patient with fibrolamellar hepatocellular carcinoma who was given ipilimumab and sequential radiation to the lung

Patient no. 4 is a 20-year-old woman with a diagnosis of fibrolamellar hepatocellular carcinoma with metastases in the lung (Fig. 2). After several treatments including chemotherapy and Y-90, metastatic lesions appeared in the lungs and were growing. The patient subsequently joined a trial of ipilimumab and sequential SABR, in which 50 Gy was given in 4 fractions to a left lung lesion (Fig. 2a). Review of the radiotherapy plan revealed that a lesion in the left lower lung had received low-dose scatter radiation (3 Gy total) (Fig. 2b) and a lesion in the right lower lobe had received no scatter dose (Fig. 2c). At 6 months after SABR, follow-up imaging showed resolution of the left lower lobe metastasis (Fig. 2b) but significant progression of the right lower lobe metastasis (Fig. 2c).

**Fig. 3:**
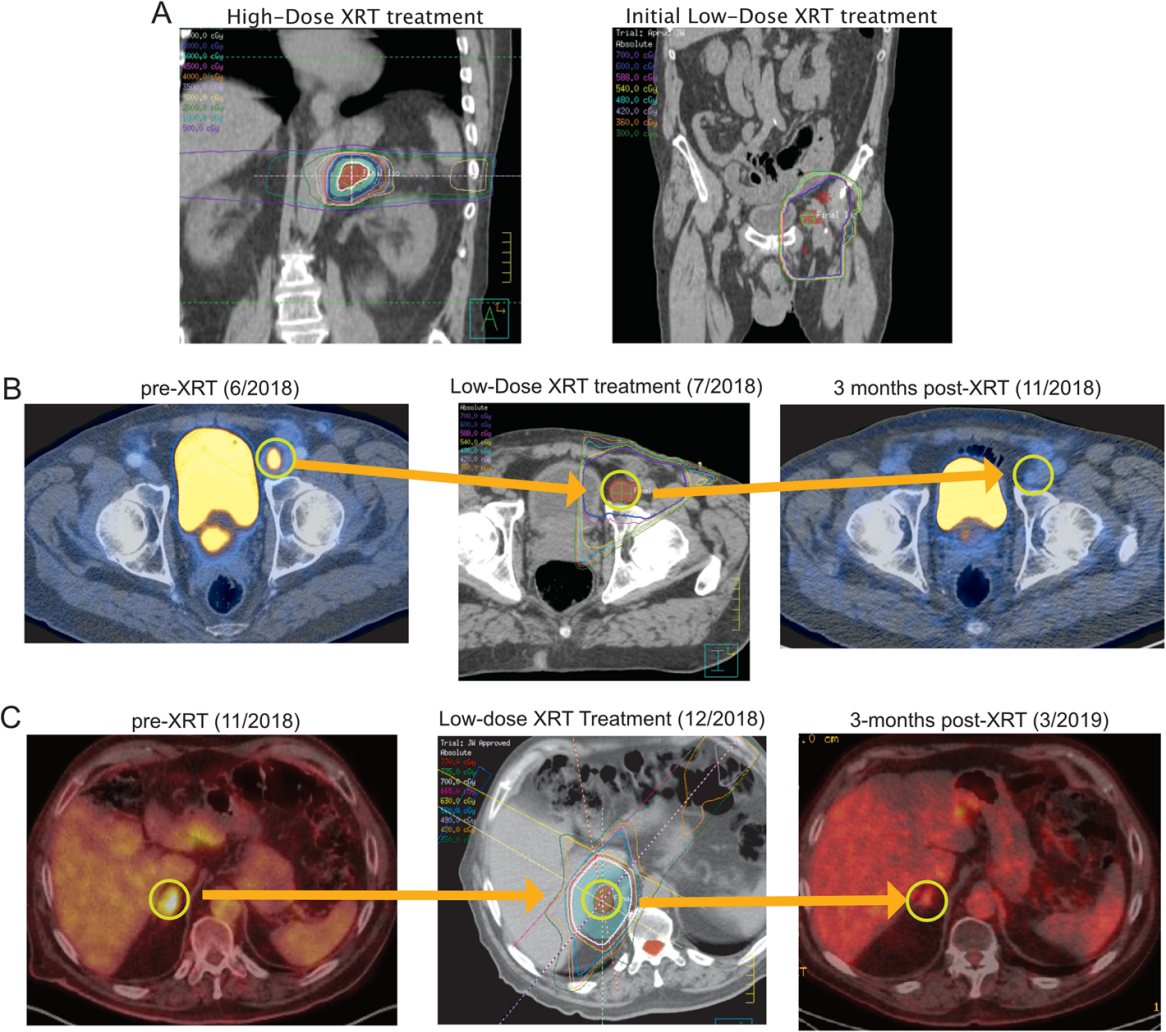
Representative scans from a patient receiving intentional low-dose radiation. Scans from a 69-year-old patient with Merkel cell carcinoma with previous disease progression on atezolizumab and bevacizumab who was given low-dose radiation to an involved inguinal node. An area receiving no radiation in the right adrenal gland developed a metastasis 3 months later, which was subsequently treated and shown to have improved radiographically as well

After observing similar responses to low-dose scatter radiation in several patients, we began to prospectively use low-dose radiation (with a separate isocenter) with immunotherapy to treat another 6 patients (Patients 21–26 in Table 1). One such patient was Patient 23, a 69-year-old man with metastatic Merkel cell carcinoma with adrenal and inguinal involvement (Fig. 3). He received 12 cycles of atezolizumab and bevacizumab before experiencing progression of the inguinal mass, at which time he was referred to radiation oncology. The adrenal mass was treated to 70 Gy in 7 fractions (Fig. 3a, *left*) and the inguinal masses to 6 Gy in 6 fractions (Fig. 3a, *right*). At a 3-month follow-up visit, CT scans showed significant improvement of the inguinal lesion and continued to maintain response (Fig. 3b). A metastatic lesion appeared in the right adrenal gland, which had received no radiation previously. This lesion was subsequently given 7 Gy in 5 fractions (with only maintenance atezolizumab in the interim) and 3 months later was found to be significantly improved radiographically (Fig. 3c).

## Discussion

To date, the rationale for using low-dose radiation (doses below the threshold thought to physically damage DNA or kill cancer cells directly) to enhance immune-cell killing in combination with immunotherapy has been largely theoretical. By evaluating patients being treated in three ongoing prospective clinical trials, and by focusing on lesions treated with low-dose radiation and entirely unirradiated lesions, this preliminary assessment suggests that lesions exposed to low-dose radiation experience clinically meaningful reductions in size relative to lesions that receive no radiation.

These results have notable implications for addressing a problem that has troubled onco-immunology for years, that is, how to turn abscopal responses from rare, inconsistent, and incidental findings to those that can be deliberately induced. Given metastatic disease remains mostly non-curable, factors that promote abscopal responses are actively being sought, as are ways to manipulate those factors in ways that reliably induce these effects in patients [7].

It is becoming increasingly apparent that the tumor stroma provides a substantially hostile environment to the antitumoral immune system, largely by means of cellular signaling and metabolic/transcriptional changes. Although manipulating the tumor stroma in efforts to enhance abscopal responses has been difficult, low-dose radiation may accomplish this by modulating the tumor stroma. Preclinical studies have shown the ability of low-dose radiation to polarize macrophages into a immunoproliferative M1 subtype, which enhances T-cell responses in this otherwise toxic tumor microenvironment [8]. Further, other findings, recently presented in abstract form [4], suggest that low-dose radiation may convert the stroma to a more favorable environment that induces homing of T lymphocytes, perhaps via reducing TGF-β signaling, which in turn results in decreased immunosuppressive cell signaling. Our findings offer a clinical proof-of-principle for this concept, given that lesions that did not receive radiation responded only if another lesion in the same patient had responded to low-dose radiation. This also suggests a potential way of inducing systemic responses by using local therapy [9].

**Fig. 4:**
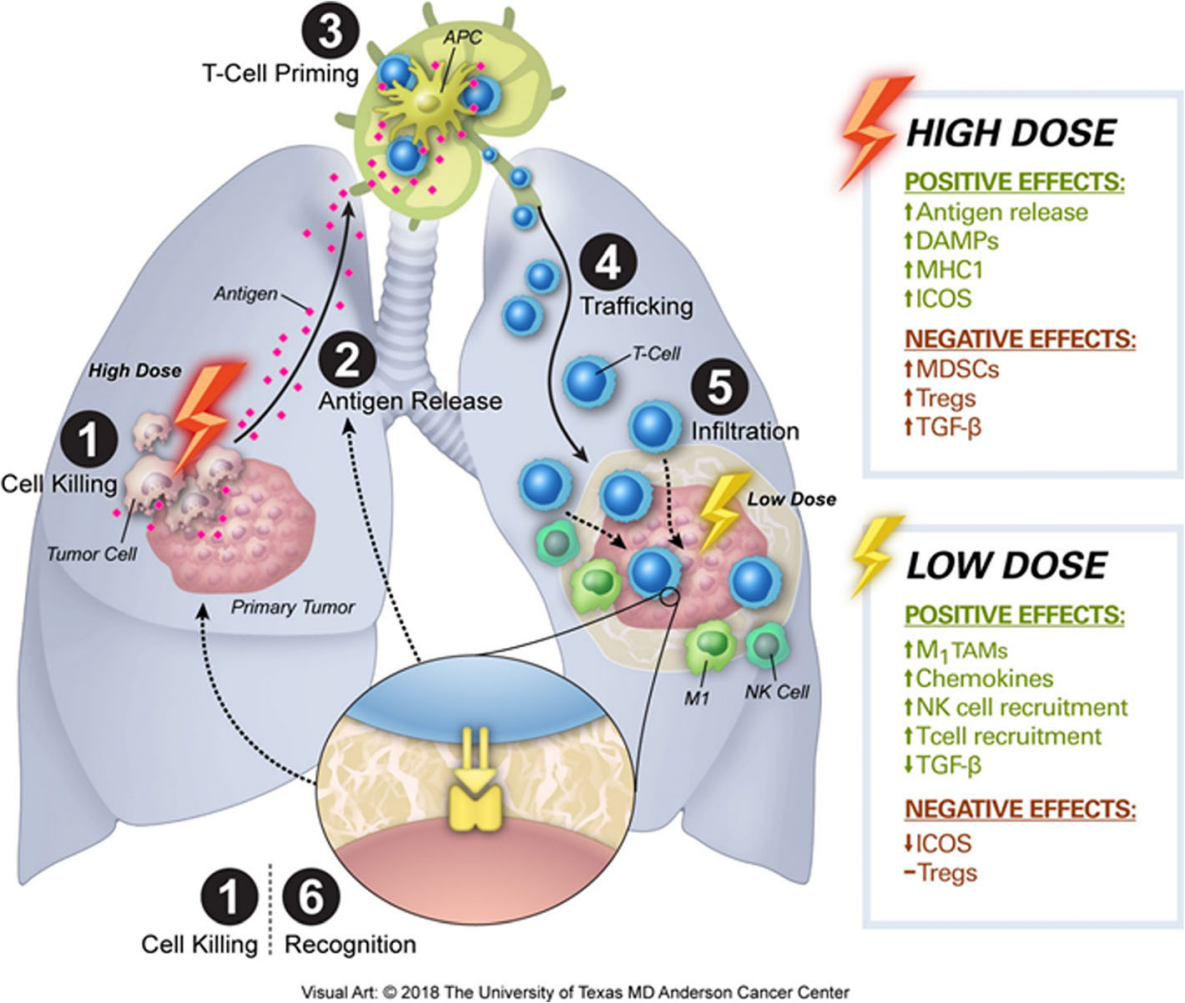
Visual representation of two uses of radiation and how low-dose radiation and high-dose radiation affect the immune cell cycle. High-dose radiation is beneficial in directly killing primary tumor cells (1), which allows antigen release (2) and leads to T-cell priming (3). Immunotherapy decreases T-cell exhaustion and enhances lymphocyte trafficking to secondary tumors (4). Low-dose radiation, by contrast, modulated the tumor stroma and enhances infiltration of natural killer (NK) cells and T cells into secondary tumor sites (5), leading to enhanced immune-cell recognition of tumor cells (6) and resulting in ongoing tumor cell killing (1) and antigen release (2). Abbreviations: DAMPs, danger-associated molecular patterns; MHC1, major histocompatibility complex 1; ICOS, the immune checkpoint ‘inducible co-stimulator’; MDSCs, myeloid-derived suppressor cells; Tregs, T regulatory cells; TGF-β, tumor growth factor-beta; TAMs, tumor-associated macrophages

This work is an integral component of the combined low-dose and high-dose radiation concept now being tested prospectively in NCT02710253, one of the three trials from which the current study dataset was derived. In this approach, high-dose radiation is given together with immune checkpoint inhibitors and with deliberate delivery of low-dose radiation, ideally to all known sites of disease. The assumption is that high-dose radiation acts to directly kill tumors, increase antigen release, and prime T cells; these newly primed T lymphocytes are further stimulated by the immunotherapeutic agents, which also prevent T-cell exhaustion. Theoretically, introducing the simultaneous delivery of low-dose radiation to other tumors throughout the body would modulate the tumor stroma throughout the body so as to facilitate infiltration of tumors by the primed T lymphocytes, which must come in direct contact with tumor cells to kill them and instigate further antigen release (Fig. 4).

In addition to corroboration of our current findings, another goal of future research should be to evaluate other factors that may facilitate or synergize with the triad of high-dose radiation, immunotherapy, and low-dose radiation. For example, elucidating the optimal timing of radiation and immunotherapy, now that the safety of these combinations has been recognized [10–12]. Low-dose radiation has been used for decades (e.g., whole-lung irradiation to 12–20 Gy for children with Ewing sarcoma) and additional FDA approvals would not be needed for this novel application [13–15]. The value of low-dose radiation for overcoming resistance to immunotherapies is also being explored in head and neck cancer in NCT03085719. Ultimately, the use of low-dose radiation could provide substantial benefit in tumor control, which is particularly relevant considering the increased toxicity and cost associated with using multiple immunotherapies at once [16–18]. Another important issue is whether tumors at different sites (e.g. lung versus liver versus bone) respond differently to low-dose radiation, or whether disease in the lymph nodes (a site of lymphocytic trafficking) responds differently from parenchymal disease.

Although a major strength of this investigation was that each patient had been treated prospectively, and some patients were deliberately treated for purposes of inducing low-dose radiation-related tumor responses, we acknowledge the shortcomings. Each treatment protocol, and the enrolled patient population was fundamentally different, leading to some degree of treatment heterogeneity. Moreover, the wide variety of disease sites and histologic subtypes may prevent uniform applicability of our findings. However, we believe a diverse study cohort is also a strength in that it shows that low-dose radiation was effective for a variety of tumor types, treatment timing, and irradiated sites. The promising patient responses in this diverse cohort prompt further studies for specific histologic subtypes. Also, this study was observational and thus causation cannot be inferred; however, issues of causation are being addressed in a dedicated prospective trial of low-dose irradiation currently underway (NCT02710253).

## Conclusions

In conclusion, this report further demonstrates the effects of low-dose radiation in combination with high-dose radiation and immunotherapy. Low-dose radiation appears to provide beneficial responses in secondary tumors and may yield durable systemic responses to immunotherapy. Further prospective investigations are warranted to evaluate the efficacy of this approach.

## Additional files

Additional file 1: **Figure S1**. Subgroup analysis to compare the response between SBRT(25Gy/5, 50Gy/4, 60Gy/10 and 70Gy/10) and hyperfraction radiaton (45Gy/15, 50Gy/20 and 52.5Gy/15). (DOCX 122 kb)

Additional file 2: **Table S1.** Mantel-Haenzel tests for independence of the variables. (DOCX 15 kb)

## Data Availability

The datasets generated and analyzed during the current study are available from the corresponding author on reasonable request.

## Abbreviations

CR: Complete response
CT: Computer tomography
IMRT: Intensity modulated radiotherapy
PD: Progressive disease
PET: Positive-emission tomography
PR: Partial response
SABR: Stereotactic ablative radiotherapy
SD: Stable disease
SD: Standard deviation
