# Supplementary material for: Influence of low-dose radiation on abscopal responses in patients receiving high-dose radiation and immunotherapy
Source: J Immunother Cancer. 2019 Sep 4;7:237. doi: 10.1186/s40425-019-0718-6 (PMC6727581; doi:10.1186/s40425-019-0718-6)
Supplement: Supplementary file 1 — Figure S1. Subgroup analysis to compare the response between SBRT(25Gy/5, 50Gy/4, 60Gy/10 and 70Gy/10) and hyperfraction radiaton (45Gy/15, 50Gy/20 and 52.5Gy/15). (DOCX 122 kb) [file 40425_2019_718_MOESM1_ESM.docx]

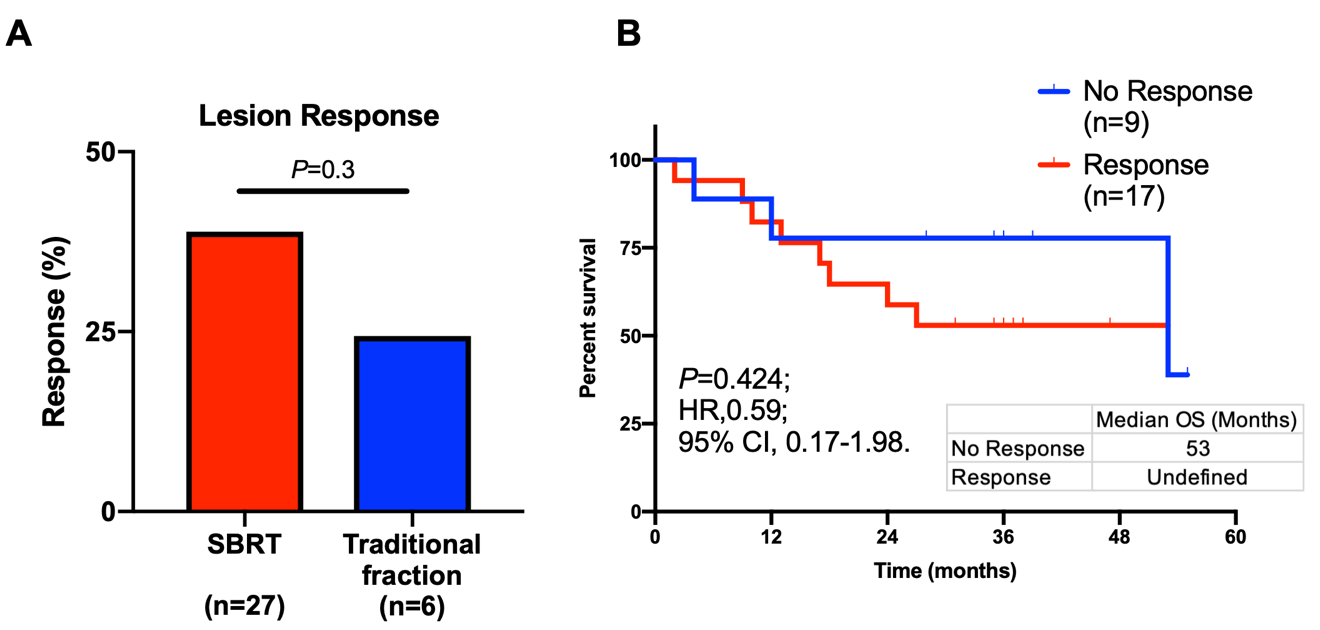


**Additional file 1: Figure S1**: subgroup analysis to compare the response between SBRT(25Gy/5, 50Gy/4, 60Gy/10 and 70Gy/10) and hyperfraction radiaton (45Gy/15, 50Gy/20 and 52.5Gy/15). No statistical difference was found for this comparison (P=0.3) (A); Overall survival between those whose low dose lesions responded and those whose did not, there was no statistical difference between these two groups (P=0.42; HR=0.59; 95% CI, 0.17-1.98)( B).
