# Supplementary material for: Influence of low-dose radiation on abscopal responses in patients receiving high-dose radiation and immunotherapy
Source: J Immunother Cancer. 2019 Sep 4;7:237. doi: 10.1186/s40425-019-0718-6 (PMC6727581; doi:10.1186/s40425-019-0718-6)
Supplement: Supplementary file 2 — Table S1. Mantel-Haenzel tests for independence of the variables. (DOCX 15 kb) [file 40425_2019_718_MOESM2_ESM.docx]

| Additional file 2: Table S1. Mantel-Haenzel tests for independence of the variables | | | |
| --- | --- | --- | --- |
|  | *P* | OR | 95% CI |
| Age |  |  |  |
| ≤65/>65 | 0.514 | 1.75 | 0.326-9.396 |
| Gender |  |  |  |
| Female/male | 0.571 | 1.602 | 0.313-8.192 |
| Immunotherapy Agent |  |  |  |
| PD1&PD-L1/CTLA4 | 0.556 | 1.645 | 0.314-8.625 |
| High-RT Site |  |  |  |
| Non-Lung/Lung | 0.606 | 1.528 | 0.305-7.652 |
| RT Fraction |  |  |  |
| Traditional fraction/SBRT | 0.866 | 1.514 | 0.22-6.053 |
| Low-Dose RT Site |  |  |  |
| Non-Lung/Lung | 0.881 | 1.143 | 0.198-6.589 |
